# The Dipeptidyl Peptidase-4 Inhibitor Des-Fluoro-Sitagliptin Regulates Brown Adipose Tissue Uncoupling Protein Levels in Mice with Diet-Induced Obesity

**DOI:** 10.1371/journal.pone.0063626

**Published:** 2013-05-16

**Authors:** Takanobu Shimasaki, Takayuki Masaki, Kimihiko Mitsutomi, Daisuke Ueno, Koro Gotoh, Seiichi Chiba, Tetsuya Kakuma, Hironobu Yoshimatsu

**Affiliations:** Department of Internal Medicine 1, Faculty of Medicine, Oita University, Yufu, Japan; Virginia Commonwealth University, United States of America

## Abstract

**Objective:**

Dipeptidyl peptidase (DPP)-4 is responsible for the degradation of several peptides that contain an alanine or proline at the penultimate position or position P1. DPP-4 inhibitors (DPP-4is) have protective effects against type-2 diabetes and several metabolic disorders.

**Methods:**

In the present study, we examined the effects of des-fluoro-sitagliptin (DFS), a DDP-4i, on body adiposity and levels of peroxisome proliferator-activated receptor (PPAR)-α, PPAR-γ coactivator-1 (PGC-1), and uncoupling proteins (UCPs) in mice with diet-induced obesity.

**Results:**

Treatment with DFS dose-dependently decreased the weight of white adipose tissue and serum levels of glucose, compared with controls, without influencing food intake (*P*<0.05). Additionally, DFS treatment increased the levels of PPAR-α, PGC-1, and UCPs in brown adipose tissue (BAT), and of PPAR-α and UCP3 in skeletal muscle (*P*<0.05). Furthermore, the effects on BAT PGC-1 and muscle PPAR-α levels were attenuated by treatment with the glucagon-like peptide 1 (GLP-1) antagonist exendin (9–39). Interestingly, hypothalamic levels of proopiomelanocortin (POMC) were increased by DFS treatment and the effects of DFS on PPAR-α, PGC-1, and UCP levels were attenuated in melanocortin (MC)-4 receptor-deficient mice.

**Conclusions:**

In conclusion, high-dose DFS appeared to regulate body adiposity and UCPs in mice with diet-induced obesity, at least partly through a GLP-1 and/or MC-4 pathway.

## Introduction

Dipeptidyl peptidase (DPP)-4 is responsible for the degradation of numerous biologically active peptides and chemokines that contain an alanine or proline at the penultimate position or position P1. A DPP-4 inhibitor (DPP-4i) acts by inhibiting the breakdown of regulatory peptides including incretins, such as glucagon-like peptide-1 (GLP-1) and glucose-dependent insulinotropic polypeptide (GIP), and increasing insulin release [1]. However, the clinical benefits of DPP-4i therapy are not fully explained by the increased insulin release alone, and other mechanisms are thought to involve effects on β-cell mass, β-cell apoptosis, and other tissues [2].

Recently, extrapancreatic actions of GLP-1 on endothelial cells and the liver have been reported [3], [4]. Additionally, effects of DPP-4i and GLP-1 on adipose tissue have been described [5], [6]. Studies performed in isolated adipocytes have demonstrated that GLP-1 has the ability to induce both lipogenic and lipolytic mechanisms in white adipose tissue (WAT) [7], [8]. These GLP-1 effects in WAT were exerted through a GLP-1-specific receptor, structurally and/or functionally distinct from that expressed in the pancreas [9]. Additionally, chronic DFS treatment decreased body weight gain in mice with diet-induced obesity [10].

In a clinical study, the combined use of DPP-4i and metformin had favorable effects on body weight in type-2 diabetic patients compared with metformin alone [11]. Additionally, almost 61% of patients showed decreased body weight when they used metformin and sitagliptin in the DURATION study [12]. These results suggest that DPP-4i influences adipose tissue and has functional roles in regulating energy metabolism as well as its anti-diabetic effects.

Generally, adipose tissue is classified into brown adipose tissue (BAT) and white adipose tissue (WAT). Uncoupling protein (UCP)-1 in BAT plays a role in energy expenditure and non-shivering thermogenesis [13], [14]. UCP2 is expressed ubiquitously in peripheral tissues, including WAT, and UCP3 is expressed primarily in skeletal muscle and adipose tissues [15]. Gene expression of these proteins is regulated by several humoral factors [16]–[18].

We hypothesized that DFS would affect adiposity and energy metabolism by modulating the expression of UCPs. However, little is known about DFS in modulating energy homeostasis. We investigated the effects of DFS on food intake, body weight, and adiposity, in addition to serum metabolic parameters, such as glucose, free fatty acids (FFAs), triglycerides, and insulin, UCP expression in peripheral tissues, O_2_ consumption, and the respiratory quotient. The goal of this study was to determine whether DFS has beneficial effects on adiposity, lipid metabolism, and energy expenditure in mice.

## Materials and Methods

### Animals

Mature male C57BL/6 mice (C57BL/6; Kbt Oriental, Fukuoka, Japan) and *Mc4r*-deficient (*Mc4r^−/−^*) mice were purchased from the Jackson Laboratory (Bar Harbor, ME, USA; stock no. 006414). Wild-type, heterozygotic, and homozygous mice were generated, and pups were genotyped by genomic polymerase chain reaction. C57BL/6 and *Mc4r^−/−^* mice were housed in a light-, temperature-, and humidity-controlled room (12/12-h light/dark cycle, lights on/off at 07∶00/19∶00 h, respectively, 21±1°C, 55±5% relative humidity). All mice were allowed free access to food and drink. The animals were fed a high-fat diet (HFD) that included 60% fat, 20% protein, and 20% carbohydrate, 5.2 kcal/g (Research Diets Inc., New Brunswick, NJ, USA). The high-fat food contained soybean oil (25/773.85 g) and lard (245/773.85). Animals were treated in accordance with the Oita University Guidelines for the Care and Use of Laboratory Animals.

### Materials

DFS (Merck & Co., Inc. Whitehouse station, NJ, USA) was mixed with HFD in food and used at a dose of 0, 0.12, 0.6, or 1.2 mg/g/day. The dose of DFS was based on both our preliminary findings and a previous report [10].

### Measuring Food intake, Body Weight, and Histological Examination

To evaluate any dose-response effect of DFS on body weight regulation, DFS mixed with HFD was administered orally at a dose of 0, 0.12, 0.6, or 1.2 mg/g/day for 4 weeks. Body weight, fat weight, serum glucose, and lipid profiles were measured in all animals at the end of the 4-week treatment period. We observed a dose-response effect of DFS and chose to use a dose of 1.2 mg/g/day.

Mice were selected and divided into DFS-treated and non-treated groups. HFD was administered for 8 weeks (from 8 to 16 weeks of age). In the DFS-treated group, DFS was administered orally at a dose of 1.2 mg/g/day for the last 4 weeks. In the control group, HFD with no DFS was given in the same way. Food intake and body weight were measured at 14∶30 h daily during the 4 weeks of treatment, and the HFD and DFS were given at 15∶00 h. Animals were euthanized 6 h after the last dose. WAT and interscapular BAT were removed and frozen in liquid nitrogen before being stored at −80°C. BAT, skeletal muscle, and epididymal WAT was dissected. The mass of body fat was measured to assess changes in body fat accumulation. The histology of epididymal WAT and levels of the UCPs were assessed in all animals at the end of the last 4 weeks of the treatment period.

To evaluate the effects of the GLP-1 receptor antagonist exendin (9–39), DFS-treated mice were injected intraperitoneally once daily for 5 d with either exendin (9–39) (25 nmol/L/kg) or saline. Each group was pair-fed.

*Mc4r^−/−^* and C57BL/6 mice were divided into DFS-treated and non-treated groups to investigate the effects of DFS on the POMC pathway. Each group was fed HFD with or without DFS at a dose of 1.2 mg/g/day and was pair-fed for 2 weeks.

### Blood Samples

We measured body weight at 14∶30 h and took blood for hormone tests at 15∶00 h. Blood was collected after a 16-h fast; serum was separated and frozen immediately at −20°C until assayed. Serum levels of glucose, insulin, triglycerides, and FFAs were measured using commercial kits (Wako Chemical, Tokyo, Japan). Serum concentrations of active GLP-1 (IBL, Tokyo, Japan), leptin (Morinaga, Tokyo, Japan), TNF-α, and IL-1β (Invitrogen, Tokyo, Japan) were measured by sandwich enzyme immunoassay using commercially available kits.

### Triglycerides in Liver and Muscle

Liver and muscle (100 mg) were homogenized in 2 mL of a solution containing 150 mM NaCl, 0.1% Triton X-100, and 10 mM Tris (pH 7.4), using a Polytron homogenizer (NS-310E; MicroTech Nichion, Chiba, Japan) for 1 min. The triglyceride content of 100 µL of this solution was determined using a commercial kit (Wako Pure Chemical, Osaka, Japan).

### Histological Analyses

Small pieces of epididymal WAT, BAT, and muscle were dissected, washed in saline, fixed in 10% formalin, and embedded in paraffin. Tissue sections were cut at a thickness of 20 µm and stained with hematoxylin and eosin. All images were captured with a Biorevo BZ-9000 microscope (Keyence, Osaka, Japan), and morphometric analyses of WAT were performed using measurement-module software (Biorevo BZ-H2A; Keyence).

### Western Blotting

Western blotting was performed as described previously [19]. Frozen tissues were homogenized in Tris buffer (pH 7.4), centrifuged, and boiled. The total protein concentration of the tissue was quantified using the Bradford method [20]. After determining the total protein concentration, an equal amount of total protein was loaded on 8% sodium dodecyl sulfate-polyacrylamide gels for electrophoresis and was then transferred electrophoretically onto a polyvinylidene difluoride membrane (Bio-Rad Laboratories, Richmond, CA, USA). Membranes were blocked with 0.4% bovine serum albumin for 5 min and then incubated for 1 h with primary antibodies at room temperature and then for 1 h at room temperature with the secondary antibody. The primary antibody solution consisted of a polyclonal antiserum (5 g/L) with specificity for UCP1 (catalog no. sc-6529), UCP2 (catalog no. sc-6526), UCP3 (catalog no. sc-7756), PPAR-α (catalog no. sc-9000), PGC-1 (catalog no. sc-13067), POMC (catalog no. sc-20148) and α-tubulin (catalog no. sc-5546) (Santa Cruz Biotechnology). Several markers were detected by enhanced chemiluminescence (Amersham Life Science, Buckinghamshire, UK) and quantitated using a ChemiDoc XRS system (Bio-Rad, Hercules, CA, USA).

### Indirect Calorimetry

*In vivo* indirect calorimetry was performed using the Oxymax system (Columbus Instruments, Columbus, OH, USA). Constant airflow (0.6 l/min) was drawn through the chamber and monitored using a flow meter. To calculate oxygen consumption (VO_2_), carbon dioxide production and respiratory quotient (RQ; ratio of carbon dioxide production to VO_2_) were monitored at the inlet and outlet of the scaled chambers. The animals were randomly placed into the experimental chambers with free access to food and water. Mice were housed individually in cages, through which air of known O_2_ concentration was passed at a constant flow rate. Calorimetry was performed after treatment with DPP-4i during light and dark phases. VO_2_ and RQ samples were collected approximately every 15 min for 24 h. For each time point, the samples for each group during the light and dark phases were averaged. The samples for each group were averaged for each time point.

### Statistical Analyses

All data are expressed as means ± SEM. We used ANOVA with a *post hoc* Fisher’s protected least significant difference test to analyze differences with multiple comparisons (StatView 5.0; SAS Institute, Cary, NC, USA), or the Mann-Whitney *U-* test, where appropriate. In the dose-dependency study, we used a simple regression test and Pearson’s coefficient test.

## Results

### Effects of DFS Treatment on Food Intake, Body Weight, WAT Weight, Serum Glucose, Insulin, FFAs, Triglycerides and Leptin in Mice with Diet-induced Obesity

**Figure 1:**
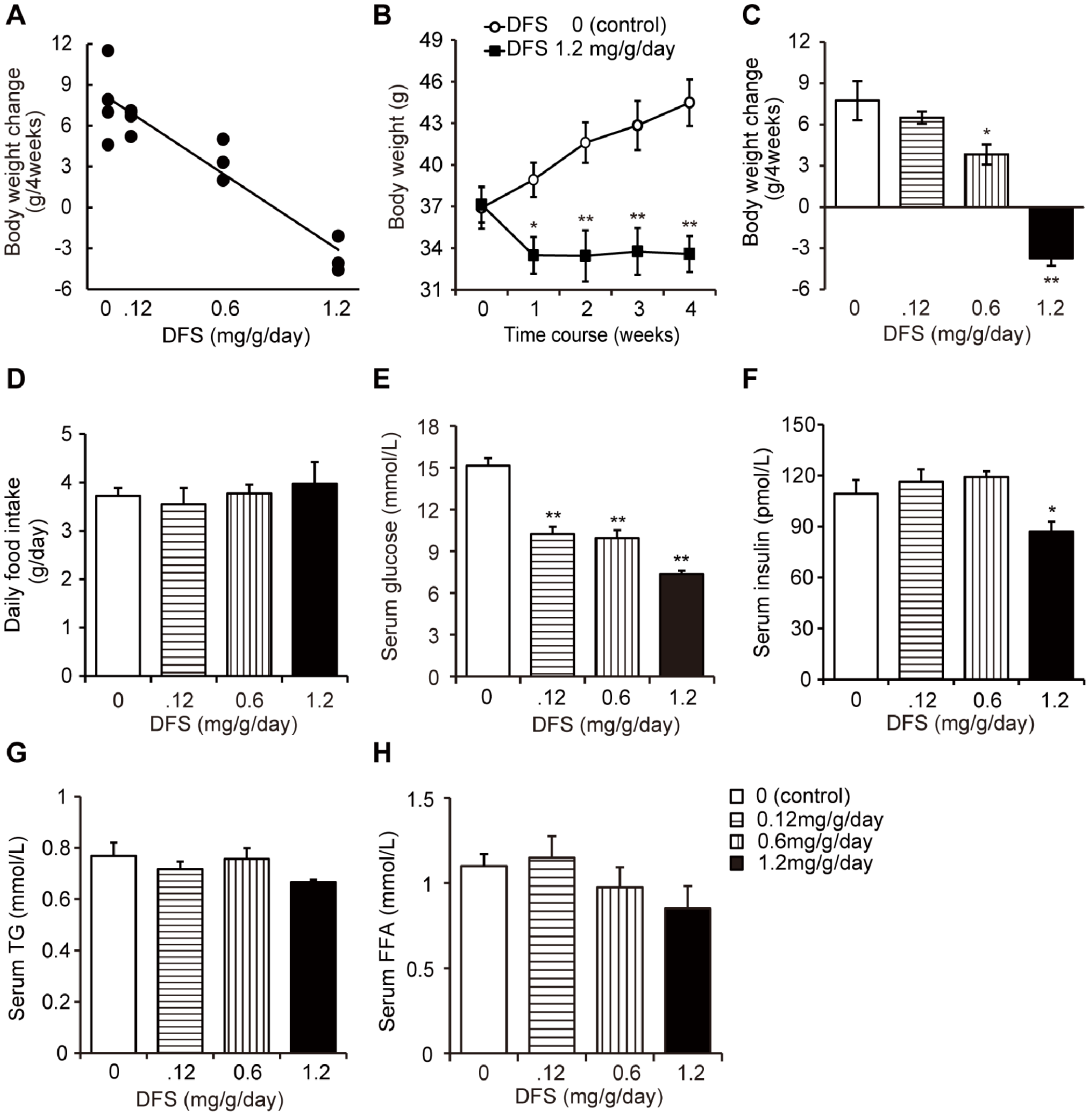
Effects of DFS in mice fed a high-fat diet (HFD mice). (A) Dose-dependent effect of DFS on change in body weight. (**B**) Time course of body weight changes in mice treated with DFS for 4 weeks and untreated mice. Black squares and solid lines = DFS 1.2 mg/g/day; white circles and solid lines = untreated controls. Effect of DFS treatment on change in body weight (**C**), average daily food intake (**D**), and serum levels of glucose (**E**), insulin (**F**), triglycerides (TGs) (**G**), and free fatty acids (FFAs) (**H**) in HFD mice over the entire 4-week treatment period. White bars = untreated controls; horizontal striped bars = DFS 0.12 mg/g/day; vertical striped bars = DFS 0.6 mg/g/day; black bars; DFS 1.2 mg/g/day. Values are the means and standard errors (n = 3–5 per group). *P<0.05, **P<0.01 *vs.* controls.

**Figure 2:**
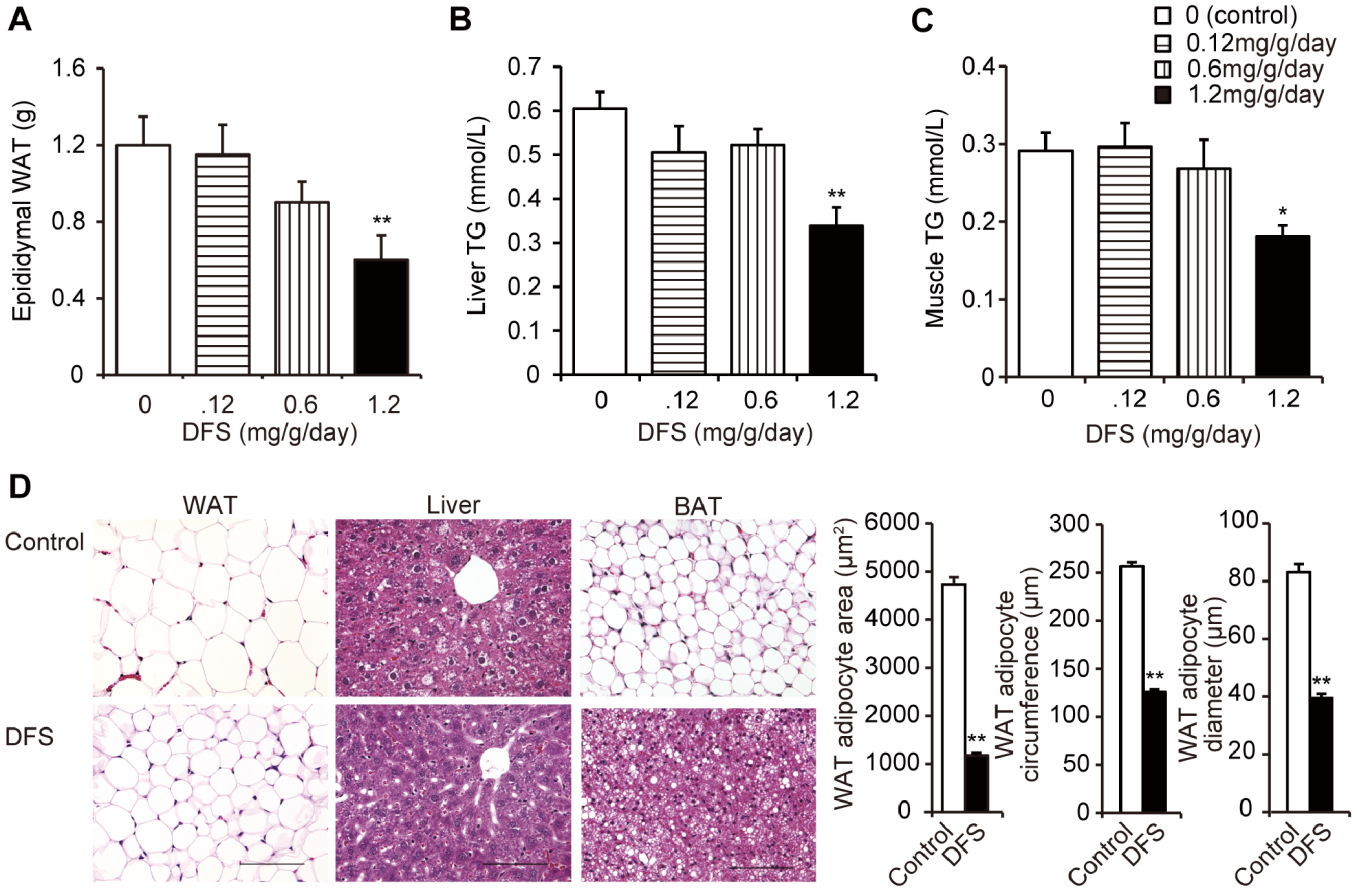
Effects of DFS on epididymal WAT, liver, muscle, and BAT in mice with diet-induced obesity. Weight of epididymal WAT (**A**) and triglyceride (TG) levels in the liver (**B**) and muscle (**C**) in HFD mice over the entire 4-week treatment period. White bars = untreated controls; horizontal striped bars = DFS 0.12 mg/g/day; vertical striped bars = DFS 0.6 mg/g/day; black bars = DFS 1.2 mg/g/day. Values are the means and standard errors (n = 4 per group). **P<0.01 and *P<0.05 *vs.* controls. (**D**) Histological analysis of epididymal WAT, liver, and BAT in DFS-treated and untreated mice. Scale bar = 100 µm. Tissues were fixed in formalin and stained with hematoxylin and eosin (H&E, ×400). White bars = untreated controls; black bars = DFS-treated mice. Values are the means and standard errors of WAT adipocyte area, circumference, and maximum diameter. **P<0.01 *vs.* the control group; for adipocytes, n = 5 per group.

Figure 1*A* shows the dose-dependent effect of DFS on body weight (DFS dose: 0, 0.12, 0.6, or 1.2 mg/g/day; *P*<0.001, r = −0.927; Fig. 1*A*). Figure 1*B* shows the time course of body weight in mice treated with DFS for 4 weeks (DFS dose: 0.12 mg/g/day) and untreated mice (*P*<0.05; Fig. 1*B*). There was no significant difference in daily food consumption between DFS-treated (DFS dose: 0.12, 0.6, or 1.2 mg/g/day) and untreated mice with diet-induced obesity, nor in body weight change between DFS-treated (DFS dose: 0.12 mg/g/day) and untreated (*P*>0.1; Fig. 1, *C* and *D*). Between DFS-treated (DFS dose: 0.6 or 1.2 mg/g/day) and untreated, significant differences did exist (*P*<0.05 for each; Fig. 1*B and C*). Additionally, serum glucose and insulin levels decreased in the DFS group (DFS dose: 1.2 mg/g/day), compared with the controls (*P*<0.01 for serum glucose; Fig. 1*E*) (*P*<0.05 for serum insulin; Fig. 1*F*). There were no significant differences in serum triglyceride or FFA levels between the DFS group and controls (*P*>0.05 for serum triglycerides; Fig. 1*G*) (*P*>0.1 for serum FFAs; Fig. 1*H*). Epididymal adiposity was attenuated in the DFS group (DFS dose: 1.2 mg/g/day) compared with controls (*P*<0.01 for epididymal WAT; Fig. 2*A*). Serum leptin levels were lower in the DFS group than in the control group (7.9±1.6 ng/mL *vs.* 13.3±2.5 ng/mL, *P*<0.05).

### Changes in the Levels of TNF-α and IL-1β after DFS Treatment

The level of circulating IL-1β, but not TNF-α, was lower in the DFS group than in the control group (22.0±0.2 pg/mL *vs.* 28.5±1.1 pg/mL, *P*<0.05). In contrast, the circulating level of IL-1β was not significantly different in the DFS-treated *Mc4r^−/−^* animals (51.3±0.5 pg/mL in DFS-treated animals *vs.* 60.5±11.7 pg/mL in controls, *P*>0.1).

### Effects of DFS Treatment on Triglyceride Content of Liver and Muscle, and Morphology of Liver and BAT

DFS treatment decreased the triglyceride contents of liver and muscle tissue, compared with controls (*P*<0.01 for liver triglycerides; Fig. 2*B*) (*P*<0.05 for muscle triglycerides; Fig. 2*C*). Figure 2*D* shows the morphology of the epididymal WAT, liver, and BAT; cell size in epididymal WAT decreased in the DFS group compared with the controls (*P*<0.01 for adipocyte area, circumference, and diameter; Fig. 2*D*).

### Effects of DFS Treatment on Oxygen Consumption and RQ

**Figure 3:**
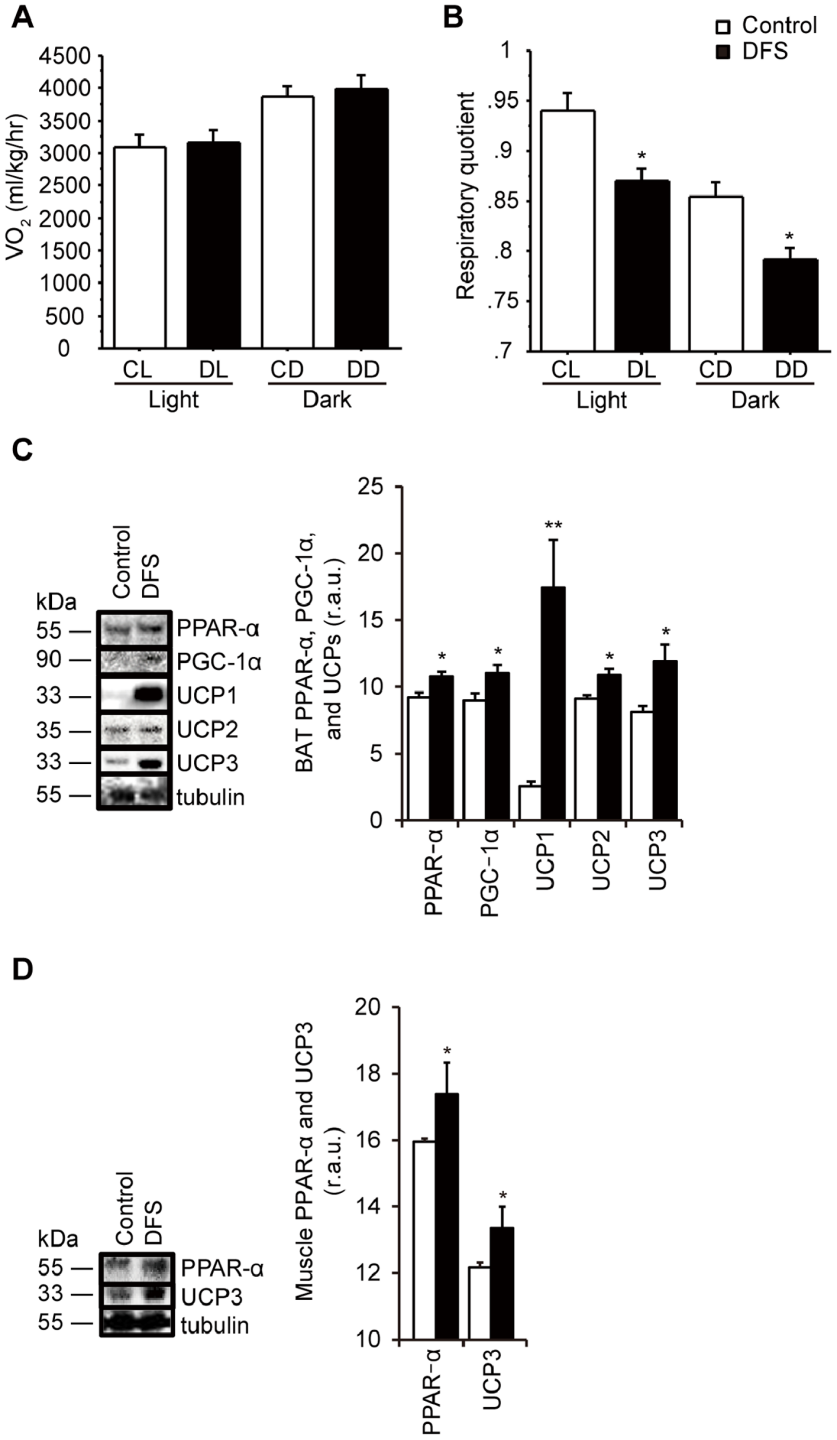
Effects of DFS on oxygen consumption, respiratory quotient, BAT, and muscle in HFD mice. (A & B) Effects of DFS on respiratory quotient (RQ) and oxygen consumption. Light phase after vehicle treatment (CL) and dark phase after vehicle treatment (CD); light phase after DFS treatment (DL) and dark phase after DFS treatment (DD). Effects of DFS on relative levels of PPAR-α, PGC-1α, and UCPs in BAT (**C**), and of PPAR-α, and UCP3 protein expression in muscle (**D**). Representative Western blots of PPAR-α, PGC-1α, and UCPs protein levels are shown. White bars = untreated controls; black bars = DFS-treated mice. Values are the means and standard errors (n = 3–5 per group). *P<0.05, **P<0.01 *vs.* controls. r.a.u., relative arbitrary unit.

Oxygen consumption and RQ were assessed pretreatment and 24 h after DFS treatment. Treatment with DFS did not increase oxygen consumption (Fig. 3*A*) but did decrease the RQ (*P*<0.05; Fig. 3*B*), compared with the controls.

### Effects of DFS on BAT PPAR-α, PGC-1α, UCPs, Muscle PPAR-α, and UCP3 Levels

Figure 3*C* shows the change in BAT PPAR-α, PGC-1α, and UCPs levels after treatment with DFS. BAT PPAR-α, PGC-1α, UCP1, UCP2, and UCP3 levels increased, by 117%, 123%, 679%, 119%, and 147%, respectively, after treatment with DFS, compared with the controls (*P*<0.05; Fig. 3*C*). No significant change occurred in WAT UCP2 levels in the DFS group, compared with controls (data not shown). Figure 3*D* shows that muscle PPAR-α and UCP3 levels were increased, by 109% and 110%, respectively, after treatment with DFS, compared with untreated mice (*P*<0.05, Fig. 3*D*).

### Effects of GLP-1 Antagonist Treatment on Body Weight and Serum Active GLP-1 Levels in DFS-treated Mice with Diet-induced Obesity

**Figure 4:**
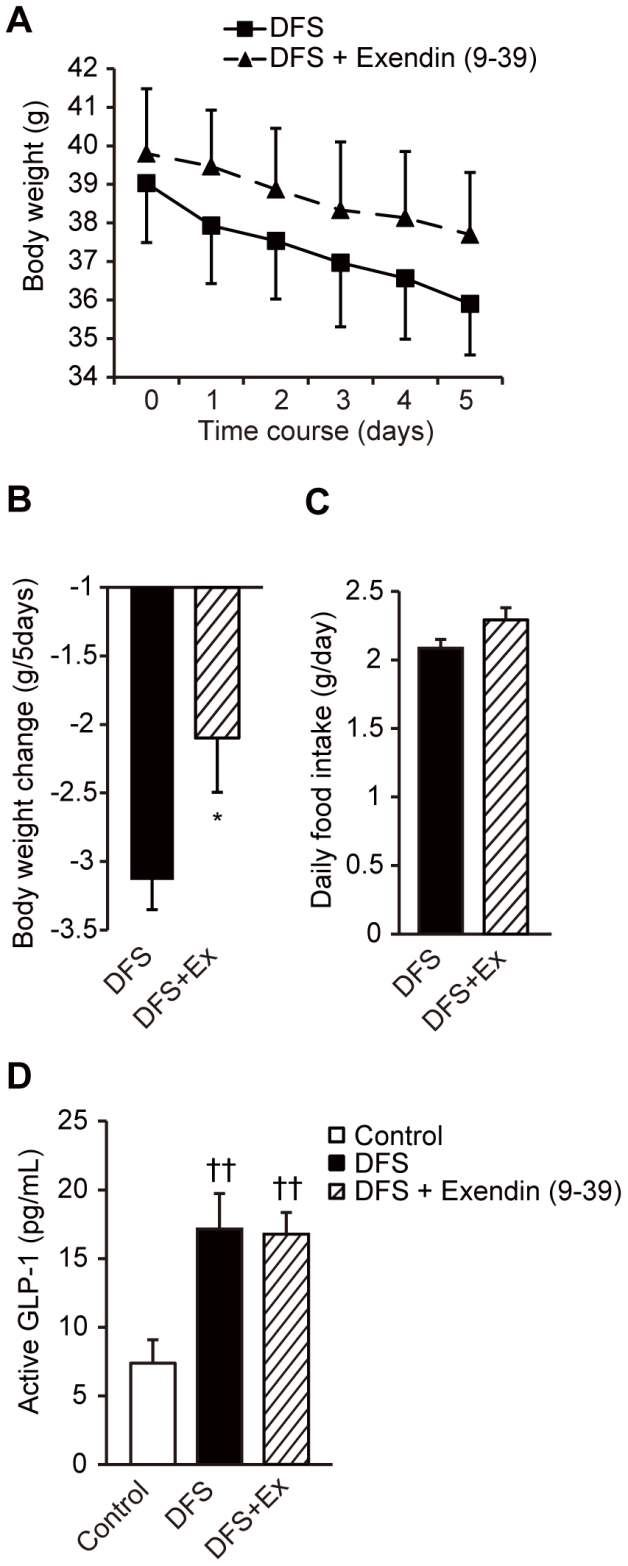
Effects of exendin (9–39) on weight and serum active GLP-1 levels in DFS-treated mice. (**A**) Body weights of saline-treated mice and mice treated with the GLP-1 receptor antagonist exendin (9–39) (Ex). Black squares and solid lines = saline-treated group; black triangles and dashed lines = Ex-treated group. (**B**) Comparison of changes in body weight at the end of the study. (**C**) Average daily food intake over the entire 5-day treatment period. Ex-treated and saline-treated mice were pair-fed. Black bars = saline-treated group; striped bars = Ex-treated group. (**D**) Serum levels of active GLP-1 in DFS-untreated controls, DFS-treated saline-treated (DFS) mice, and DFS-treated Ex-treated (DFS+Ex) mice. White bars = control; black bars = DFS; striped bars = DFS+Ex. Values are the means and standard errors (n = 3–4 per group). *P<0.05 *vs.* the DFS group, **††** *P*<0.01 *vs.* the control group.

The effect of DFS on body weight was partially attenuated by the GLP-1 antagonist exendin (9–39) compared with saline-treated mice (−2.1±0.4 g/5 days in exendin (9–39)-treated mice *vs.* −3.1±0.2 g/5 days in saline-treated mice, *P<*0.05 for body weight change; Fig. 4, *A* and *B*). There was no significant difference in daily food intake (Fig. 4*C*) or serum active GLP-1 levels (16.8±1.6 pg/mL in exendin (9–39)-treated mice *vs.* 17.1±2.6 pg/mL in saline-treated mice, *P*>0.1; Fig. 4*D*) between exendin (9–39)- and saline-treated mice. However, treatment with DFS elevated the serum active GLP-1 level almost 2.3-fold compared with controls (17.1±2.6 pg/mL in DFS-treated mice *vs.* 7.4±1.7 pg/mL in controls, *P*<0.01; Fig. 4*D*).

### Effects of DFS on BAT PGC-1α, UCP1, UCP2, Muscle PPAR-α, and UCP3 Levels in Animals Treated with a GLP-1 Antagonist

**Figure 5:**
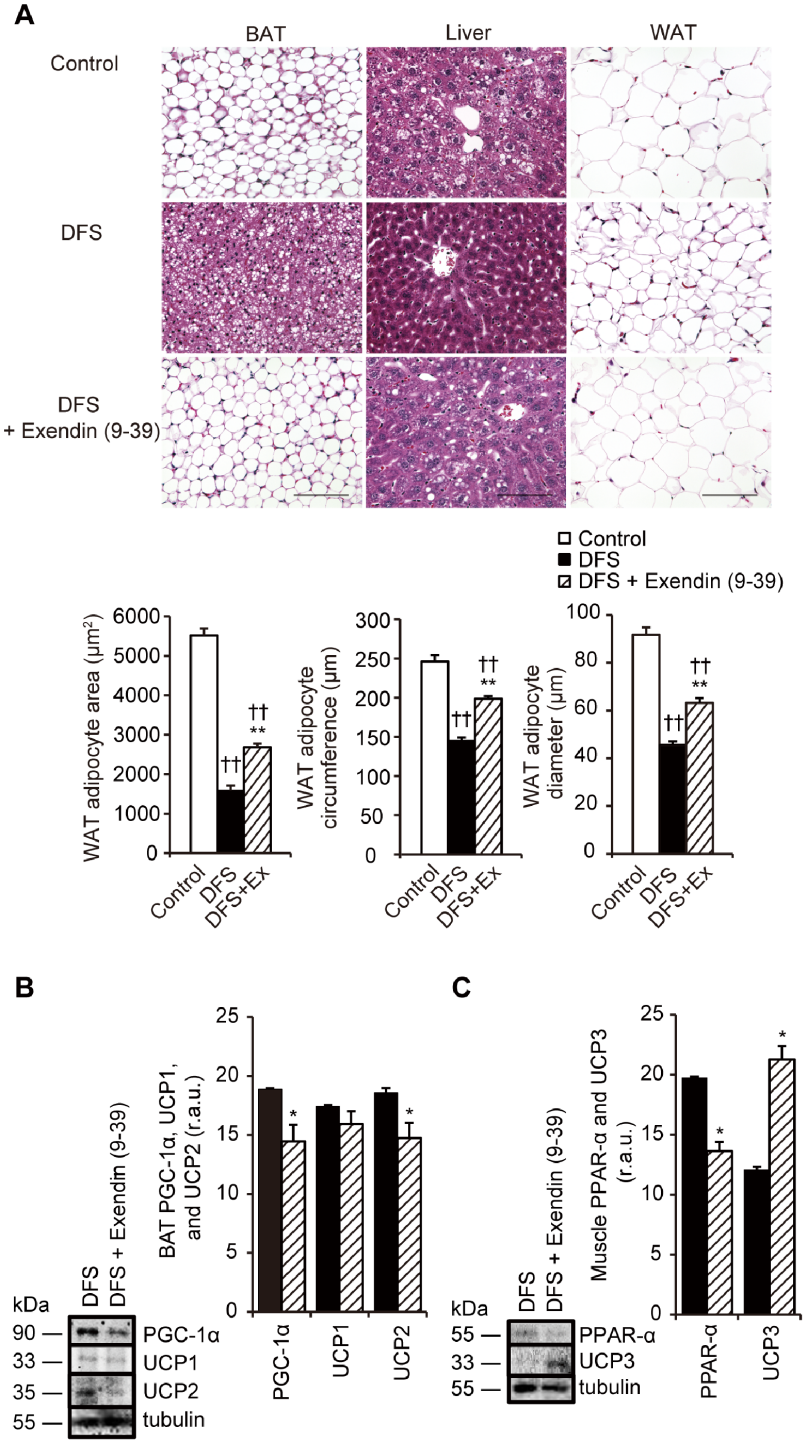
Effects of exendin (9–39) on BAT, liver, WAT, and muscle in DFS-treated mice. (A) Histological analysis of BAT, liver, and epididymal WAT in three groups of mice: untreated mice (controls), mice treated with DFS plus saline (DFS group), and mice treated with DFS plus Ex (DFS+Ex group). Scale bar = 100 µm. Tissues were fixed in formalin and stained with hematoxylin and eosin (H&E, ×400). Values are the means and standard errors of WAT adipocyte area, circumference, and maximum diameter. **P<0.01 *vs.* the DFS group, **††** *P*<0.01 *vs.* the control group; for adipocytes, n = 5 per group. Effects of Ex on BAT (**B**) and muscle (**C**) in DFS-treated mice. Representative Western blots of PPAR-α, PGC-1α, and UCP protein levels are shown. White bars = control group; black bars = DFS group; striped bars = DFS+Ex group. Values are the means and standard errors (n = 3 per group). *P<0.05 *vs.* the DFS group. r.a.u., relative arbitrary unit.

Figure 5*A* shows the morphology of the BAT, liver, and epididymal WAT; cell size in epididymal WAT increased in the DFS group treated with the GLP-1 antagonist exendin (9–39) compared to the saline-treated DFS group (*P*<0.01 for adipocyte area, circumference, and diameter; Fig. 5*A*). The effects of DFS on BAT PGC-1α, UCP2, and muscle PPAR-α levels were partly attenuated by the GLP-1 antagonist (*P*<0.05 for BAT PGC-1α, BAT UCP2, and muscle PPAR-α; Fig. 5, *B* and *C*). In contrast, the muscle UCP3 level was increased by 176% by the GLP-1 antagonist compared with the saline-treated DFS group (*P*<0.05 for muscle UCP3; Fig. 5*C*). There was no significant change in the effect of DFS on the BAT UCP1 level in mice treated with the GLP-1 antagonist (*P*>0.1 for BAT UCP1; Fig. 5*B*).

### Effects of DFS Treatment on Hypothalamic POMC Protein Levels in B57BL/6 Mice, and on Body Weight and WAT Weight in *Mc4r^−/−^* Mice

**Figure 6:**
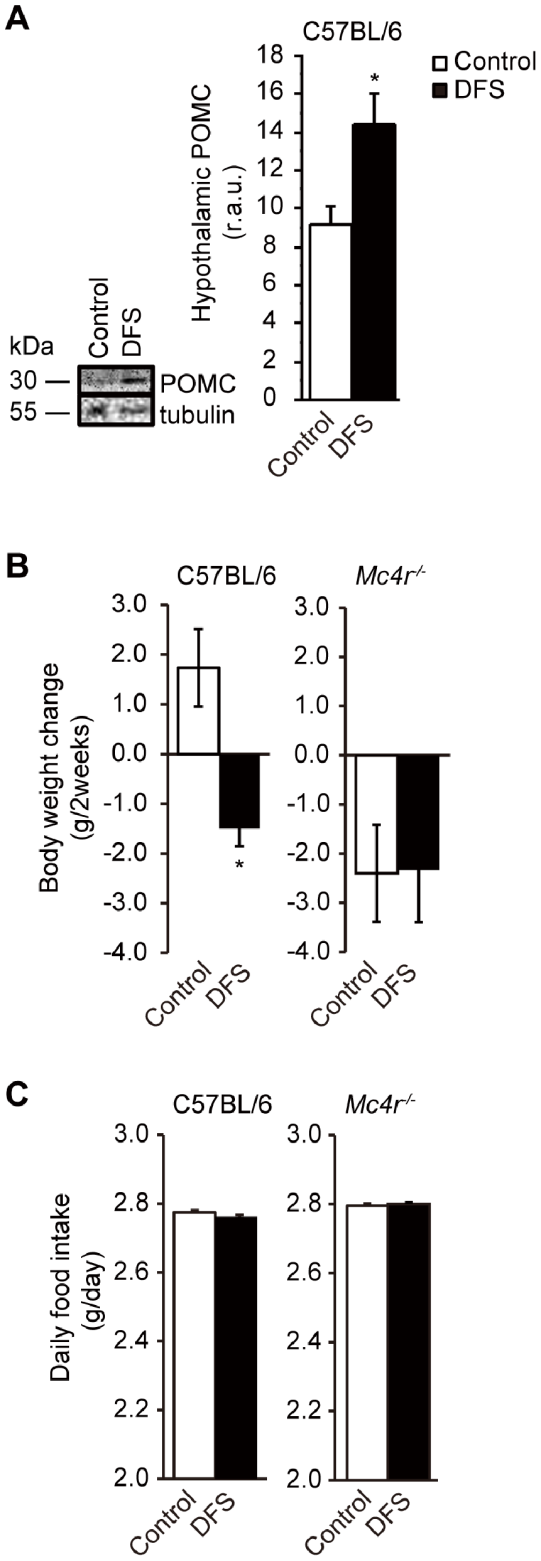
Effects of DFS on weight in *Mc4r^−/−^* mice and on hypothalamic POMC protein levels in C57BL/6 mice. (A) Changes in hypothalamic POMC protein levels after DFS treatment in C57BL/6 mice. (**B**) Changes in body weight in C57BL/6 and *Mc4r^−/−^* mice. (**C**) Average daily food intake over the entire 2-week treatment period. C57BL/6 and *Mc4r^−/−^* mice were pair-fed. White bars = untreated mice (control group); black bars = DFS-treated mice (DFS group). Values are the means and standard errors (n = 3–4 per group). *P<0.05 *vs.* controls. r.a.u., relative arbitrary unit.

DFS treatment increased the hypothalamic POMC protein level compared to controls (14.6±1.5 r.a.u. in DFS-treated mice *vs.* 9.2±0.9 r.a.u. in controls, *P*<0.05; Fig. 6*A*). Body weight and epididymal adiposity were attenuated in the DFS-treated C57BL/6 mice compared to untreated mice (*P*<0.05 for body weight change; Fig. 6*B*). There were no significant differences in body weight and epididymal adiposity between DFS-treated and non-treated *Mc4r^−/−^* mice (*P*>0.1 for body weight change; Fig. 6*B*) (epididymal WAT: data not shown). Each group was pair-fed for 2 weeks (Fig. 6*C*).

The effects of DFS on body weight and WAT weight were not observed in *Mc4r^−/−^* mice.

### Effects of DFS on BAT PPAR-α, PGC-1α, UCPs, Muscle PPAR-α, and UCP3 Levels in *Mc4r^−/−^* Mice

**Figure 7:**
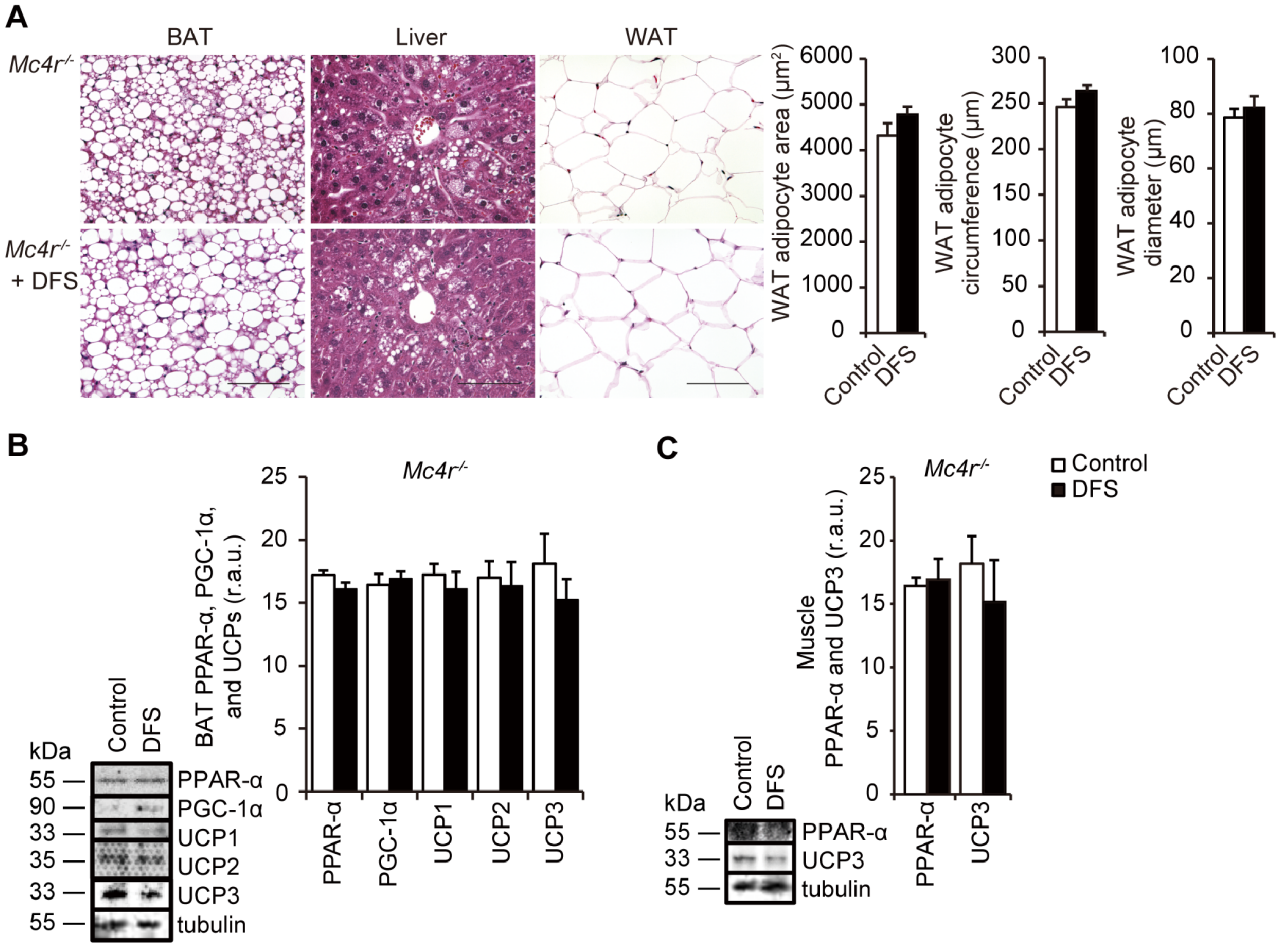
Effects of DFS on BAT, liver, WAT, and muscle in *Mc4r^−/−^* mice. (A) Histological analysis of BAT, liver, and epididymal WAT in DPP-4i des-fluoro-sitagliptin (DFS)-treated and non-treated *Mc4r^−/−^* mice. Scale bar = 100 µm. Tissues were fixed in formalin and stained with hematoxylin and eosin (H&E, ×400). Values are the means and standard errors of WAT adipocyte area and circumference and maximum diameter of adipocytes (n = 5 per group). Effects of DFS on BAT (**B**) and muscle (**C**) in *Mc4r^−/−^* mice. Representative Western blots of PPAR-α, PGC-1α, and UCP protein levels are shown. White bars = untreated mice (control group); black bars = DFS-treated mice (DFS group). Values are the means and standard errors (n = 3 per group). r.a.u., relative arbitrary unit.

Figure 7*A* shows the morphologies of the BAT, liver, and epididymal WAT; there was no significant change in cell size in epididymal WAT in the DFS group, compared to the control group (*P*>0.1 for adipocyte area, circumference, and diameter; Fig. 7*A*). The effects of DFS on BAT PPAR-α, PGC-1α, UCPs, muscle PPAR-α, and UCP3 levels were partially attenuated in *Mc4r^−/−^* mice (*P*>0.1 for BAT UCP1 and muscle UCP3; Fig. 7, *B* and *C*).

## Discussion

This study demonstrated that DFS attenuated body adiposity, without affecting food intake, in C57BL/6 mice with diet-induced obesity. Additionally, DFS treatment dose-dependently decreased body weight gain.

We examined how DFS reduced body adiposity; our results demonstrated that the administration of DFS did not affect food intake. Given this, the effect of DFS on energy metabolism may be an important factor in the DFS-induced reduction in adiposity. UCP is an inner mitochondrial membrane transporter of FFAs that dissipates the proton gradient by releasing stored energy, as heat [21]. UCP1 in BAT plays a crucial role in regulating energy expenditure and thermogenesis in rodents and the mammalian neonates, including humans [22]. Our study demonstrated that DFS treatment increased BAT UCP1 levels, reflecting a DFS-induced increase in energy expenditure. In addition to the change in BAT UCP1, in this study, DFS treatment increased muscle UCP3 protein expression. The functional meaning of this is currently unclear. It has been suggested that UCP3 contributes to the export of fatty acids from the mitochondrial matrix, rather than the regulation of energy expenditure [23]. The export of fatty acids from the mitochondrial matrix by UCP3 may prevent the accumulation of fatty acids in mitochondria and help to maintain muscular fat oxidative capacity. The DFS-induced up-regulation of muscle UCP3 might also contribute to regulating fatty acid mobilization and utilization. In this study, we confirmed the functional role of the DFS-induced increase in UCP1 protein level in BAT using calorimetry. In fact, DFS treatment did not change O_2_ consumption but decreased the respiratory quotient. This suggests that high-dose DFS treatment decreases the use of carbohydrates and increases the use of fat, which may also contribute to the reduction in body fat.

DPP-4is stabilize postprandial levels of bioactive GLP-1 and GIP [2] and have been approved for the treatment of type 2 diabetes. DPP-4is exert their actions predominantly through potentiation of GLP-1 signaling [10]. A possible involvement of DFS in regulating adiposity has been suggested: perhaps a direct action of DFS on GLP-1 levels [24], [25]. Indeed, in the present study, the effects of DFS on body weight, and UCPs were partially attenuated with a GLP-1 antagonist. It is also possible that other factors mediate the accelerating effect of DFS on UCP1. We cannot exclude the possible involvement of the central nervous system because BAT UCP1 is regulated by the hypothalamus and brainstem, through activation of the sympathetic nervous system [26]–[28]. In fact, POMC levels were increased by DFS treatment. Furthermore, the effects of DFS on body weight, WAT weight, and UCPs were attenuated in *Mc4r^−/−^* mice. HFD-induced obesity in mice increases hyperleptinemia and hypothalamic leptin resistance through induction of suppressor of cytokine signaling (SOCS)-3 [29]. Increased SOCS-3 expression in POMC neurons inhibits activation of signal transducer and activator of transcription (STAT)-3 and results in hyperphasia and obesity [30]. Several researchers have clinically investigated the anti-inflammatory and SOCS-3 suppressive effects of sitagliptin and exenatide, a GLP-1 receptor agonist [31], [32]. In the present study, treatment with DFS elevated serum levels of active GLP-1 compared with controls. In addition, GLP-1 antagonist treatment attenuated the DFS-induced body weight decrease. This suggests that the effects of DFS are partly dependent on an increase in the GLP-1 concentration.

This study demonstrated that DFS attenuated body adiposity, without affecting food intake, suggesting DFS effectively accelerated energy expenditure through a GLP-1 and/or MC-4 pathway. Taken together, DFS appears to regulate body adiposity and UCPs in mice with diet-induced obesity, at least partly through a GLP-1 and/or MC-4 pathway.

Somewhat surprising was the observation that mice treated with DFS also exhibited attenuation of weight gain on a high-fat diet. This is in contrast to a clinical study of sitagliptin. There, sitagliptin monotherapy demonstrated significant reductions in HbA1c; however, it had a neutral effect on body weight relative to baseline [33]. Continuous administration of DFS in the food in our study probably achieves more potent and sustained 24-h inhibition of DPP-4 activity relative to the twice-daily administration of vildagliptin used in previous experiments [34]. These findings may be explained in part by species-specific differences in energy homeostasis, arising as a result of loss of DPP-4 activity. Clinically, the combined use of DPP-4i and metformin has favorable effects on body weight in type-2 diabetic patients compared with that of a sulfonylurea and metformin [35]. Additionally, the combined use of pioglitazone and sitagliptin did not significantly worsen pioglitazone-induced body weight gain [36]. Furthermore, almost 61% patients showed decreased body weight when they used metformin and sitagliptin in the DURATION study [12]. The results indicated that DPP-4i may regulate body adiposity under the specific conditions that accelerated the effects of DPP-4i or combined other nutritional/environmental factors. Indeed, the effects of DPP-4i are not observed with a normal diet (unpublished data).

The present study has several limitations. First, the dose is relatively high compared with human doses. It may thus be worthwhile to investigate similarities and dissimilarities between humans and rodents. Second, the involvement of weight loss in other major organs, such as the liver, remains unresolved. Third, we did not determine the peripheral effects of DFS on energy homeostasis, including mitochondrial counts, in BAT. Further studies are necessary to address in more detail the mechanism(s) by which DFS affects energy metabolism.

In conclusion, DFS appears to regulate body adiposity and energy expenditure in mice with diet-induced obesity, partly through GLP-1 and/or MC-4 pathways.

The English in this document has been checked by at least two professional editors, both native speakers of English. For a certificate, please see: http://www.textcheck.com/certificate/NeMmt9.
